# Linzagolix versus leuprorelin in Japanese women with uterine leiomyomas: a phase 3, randomized, active-controlled, non-inferiority trial

**DOI:** 10.1093/humrep/deag106

**Published:** 2026-07-06

**Authors:** Yutaka Osuga, Tasuku Harada, Hideomi Takahashi, Asuka Kawai, Fumiya Shimamura, KLH2301 Investigators, Futoshi Arakane, Masao Fukuhara, Hideki Hanashi, Akitoshi Hasegawa, Masaki Hashimoto, Mariko Hatta, Jun Hayakawa, Kozo Hirai, Nobuyuki Hosoi, Shinya Iwasaki, Takayuki Kamiya, Yuji Kashiwazaki, Fuminori Kitada, Takashi Kitanaka, Masanori Maruyama, Ryuji Nagai, Sonoe Nishiguchi, Akira Nishikawa, Shunji Nojima, Akinori Oki, Hisato Oku, Masatoshi Sano, Mari Sawada, Yukari Sumi, Akiko Tanabe, Chisei Tei, Mizue Teramoto, Ruriko Tsushima, Rikako Uchida, Kenji Umayahara, Mamoru Urabe, Yukari Utsugisawa, Akiyoshi Yamanaka, Teruko Yasuda, Shohei Yoshida, Masuo Yoshioka, Sumie Yukawa

**Affiliations:** Department of Obstetrics and Gynecology, Graduate School of Medicine, The University of Tokyo, Tokyo, Japan; Department of Obstetrics and Gynecology, Faculty of Medicine, Tottori University, Tottori, Japan; Clinical Research Department, Kissei Pharmaceutical Co. Ltd., Tokyo, Japan; Clinical Projects Management Department, Kissei Pharmaceutical Co. Ltd., Tokyo, Japan; Clinical Research Department, Kissei Pharmaceutical Co. Ltd., Tokyo, Japan

**Keywords:** gonadotropin-releasing hormone receptor antagonist, heavy menstrual bleeding, linzagolix, randomized controlled trial, uterine leiomyomas

## Abstract

**STUDY QUESTION:**

How does the efficacy of linzagolix (a gonadotropin-releasing hormone [GnRH] receptor antagonist) compare with leuprorelin (a GnRH agonist) for the treatment of heavy menstrual bleeding (HMB) in patients with uterine leiomyomas?

**SUMMARY ANSWER:**

Reductions in menstrual bleeding with linzagolix were rapidly achieved, non-inferior to leuprorelin at Weeks 6–12, and maintained over 24 weeks of treatment.

**WHAT IS KNOWN ALREADY:**

GnRH agonists are commonly used to manage the symptoms of uterine leiomyomas; however, they are also associated with bone mineral density (BMD) loss, an initial worsening of symptoms (flare-up effect), and delayed pituitary recovery after treatment cessation. Linzagolix is a GnRH receptor antagonist that may overcome the limitations of GnRH agonists, though comparative safety and efficacy have not yet been evaluated.

**STUDY DESIGN, SIZE, DURATION:**

This was a phase 3, active-controlled, multicentre, randomized, double-blind, parallel-group, non-inferiority study, conducted in Japan between October 2022 and August 2024. In total, 287 patients were randomized 1:1 to oral linzagolix 200 mg once daily (plus leuprorelin placebo; n = 143) or subcutaneous leuprorelin 1.88 mg every 4 weeks (plus linzagolix placebo; n = 144) for 24 weeks. Patients were randomized using an interactive web response system, stratified by the presence of pain symptoms.

**PARTICIPANTS/MATERIALS, SETTING, METHODS:**

Eligible patients were premenopausal women (aged ≥20 years) with uterine leiomyomas and HMB. Key assessments throughout the treatment period included menstrual bleeding (measured using the Pictorial Blood Loss Assessment Chart [PBAC]), pain (measured using a numeric rating scale), blood haemoglobin, myoma and uterine volumes (measured by transvaginal ultrasound), patient-reported symptom severity and quality of life, plasma oestradiol, adverse events (AEs), and BMD. At the end of treatment, patients entered a follow-up period of up to 24 weeks, during which plasma oestradiol, BMD, and menstrual recovery were assessed. The primary endpoint was the proportion of patients with a total PBAC score <10 from Weeks 6 to 12 of the treatment period (non-inferiority margin, −15%).

**MAIN RESULTS AND THE ROLE OF CHANCE:**

From Weeks 6 to 12, the proportion of patients with a total PBAC score <10 was 89.9% (95% CI, 83.7, 94.4) in the linzagolix group, which was non-inferior to 90.8% (84.7, 95.0) in the leuprorelin group (difference, −0.9% [−8.6, 6.9]). Reductions in menstrual bleeding were rapidly achieved with linzagolix; median time to a total PBAC score <10 was significantly shorter in the linzagolix group versus leuprorelin (6.0 vs 20.0 days; *P *= 0.023). Improvements in pain, haemoglobin, myoma/uterine volumes, and patient-reported outcomes were comparable between groups over 24 weeks, with earlier reductions in pain and myoma/uterine volumes observed in the linzagolix group. Mean plasma oestradiol levels were rapidly reduced to <20 pg/ml by Week 2 in the linzagolix group (without the flare-up effect seen in the leuprorelin group) and were maintained through Week 24. The incidence of AEs was comparable between the linzagolix and leuprorelin groups (97.2% and 96.5%, respectively). The most common AE was hot flush in the linzagolix group (53.8%) and breakthrough bleeding in the leuprorelin group (55.6%), and the incidence of these events was highest during the first 28 days of treatment. Following the end of study treatment, mean plasma oestradiol levels tended to recover earlier in the linzagolix group versus leuprorelin, which coincided with earlier recovery of hot flush AEs, reductions in BMD, and menstruation.

**LIMITATIONS, REASONS FOR CAUTION:**

Further studies are warranted to compare linzagolix with other GnRH receptor antagonists, and to evaluate the longer-term safety and efficacy of linzagolix, with or without hormonal add-back therapy, in Japanese women with uterine leiomyomas and HMB.

**WIDER IMPLICATIONS OF THE FINDINGS:**

This phase 3 study met its primary endpoint, and demonstrated the 24-week efficacy, safety, and pharmacodynamic effects of linzagolix in Japanese women with symptomatic uterine leiomyomas. While linzagolix offered comparable efficacy to leuprorelin over 24 weeks of treatment, its earlier onset of action may make linzagolix a favourable treatment option. In particular, rapid reductions in myoma and uterine volumes suggest that linzagolix may be a useful preoperative treatment. Moreover, earlier recovery of oestradiol levels and menstruation following linzagolix may be an important consideration for women planning pregnancy after treatment. Overall, these findings support the use of linzagolix over 24 weeks for the signs and symptoms of uterine leiomyomas.

**STUDY FUNDING/COMPETING INTEREST(S):**

Funding for the study and third-party medical writing support was provided by Kissei Pharmaceutical Co., Ltd. The study sponsor participated in the design of the study; the collection, analysis, and interpretation of data; and the development of the manuscript. Y.O. and T.H. report medical writing and clinical trial advisory fees from Kissei Pharmaceutical Co., Ltd. H.T. reports patent applications with Kissei Pharmaceutical Co., Ltd. H.T., A.K., and F.S. are employees of Kissei Pharmaceutical Co., Ltd.

**TRIAL REGISTRATION NUMBER:**

NCT05440383.

**TRIAL REGISTRATION DATE:**

27 June 2022.

**DATE OF FIRST PATIENT’S ENROLMENT:**

11 October 2022.

## Introduction

Uterine leiomyomas are non-cancerous tumours that are growth-responsive to gonadal steroid hormones (i.e. oestrogen and progesterone), and occur in up to 70–80% of women (Baird *et al.*, 2003; Stewart *et al.*, 2016, 2017), though prevalence and diagnosis rates vary widely across racial and ethnic groups (Katon *et al.*, 2023; Mitro *et al.*, 2025). In Japan, 5.5 million prevalent cases of uterine leiomyomas were estimated in 2019, equating to an age-standardized rate of 8445 cases per 100,000 women (Lou *et al.*, 2023). While histologically benign and often asymptomatic, uterine leiomyomas are nevertheless a burdensome gynaecological condition for those who present with symptoms; commonly heavy menstrual bleeding (HMB) and associated anaemia, abnormal uterine bleeding, pelvic pressure and pain, and infertility (Stewart *et al.*, 2016; Lou *et al.*, 2023). These symptoms frequently interfere with daily activities and social functioning, and can greatly impair health-related quality of life (HRQoL) (Fortin *et al.*, 2018; Go *et al.*, 2020).

For patients with symptomatic uterine leiomyomas, treatment options currently include surgery or medical therapies, depending on the size, number, and location of myomas, the severity of symptoms, and the desire to have children (Stewart *et al.*, 2016; Mension *et al.*, 2024). Among medical therapies, gonadotropin-releasing hormone (GnRH) agonists (e.g. leuprorelin) suppress oestrogen and progesterone production via GnRH receptor desensitization and downregulation, thereby improving leiomyoma-related symptoms (Donnez *et al.*, 2012; Osuga *et al.*, 2019, 2021). However, GnRH agonists are generally only used as a short-term bridge to surgery, due to the risk of bone mineral density (BMD) loss resulting from hypo-oestrogenism (Stewart *et al.*, 2016; Ciebiera *et al.*, 2023; Mension *et al.*, 2024). They have also been associated with an initial worsening of symptoms caused by a transient increase in gonadotropin secretion (known as the ‘flare-up effect’), and delayed pituitary recovery after treatment cessation (Stewart *et al.*, 2016; Ciebiera *et al.*, 2023; Mension *et al.*, 2024).

Linzagolix is an oral GnRH receptor antagonist, representing a newer class of endocrine therapy that may overcome some of the limitations of GnRH agonists (Ciebiera *et al.*, 2023). By directly inhibiting pituitary GnRH receptors and gonadotropin secretion, linzagolix has been shown to rapidly reduce gonadal hormone levels and avoid the flare-up effect seen with GnRH agonists (Pohl *et al.*, 2018). The safety and efficacy of linzagolix in uterine leiomyomas and endometriosis were recently demonstrated in three pivotal phase 3, placebo-controlled studies (Donnez *et al.*, 2022, 2024). Based on these data, linzagolix was first approved in the European Union for moderate-to-severe symptoms of uterine leiomyomas in June 2022, followed by its approval for the symptomatic treatment of endometriosis in November 2024 (Keam, 2022; European Medicines Agency, 2025). In parallel with another phase 3 study (described elsewhere), herein we present the first active comparator-controlled study to evaluate the 24-week efficacy, safety, and pharmacodynamic effects of linzagolix in Japanese patients with uterine leiomyomas and HMB.

## Materials and methods

### Study design

KLH2301 was a phase 3, active-controlled, randomized, double-blind, parallel-group, non-inferiority study, conducted at 38 clinical sites in Japan between October 2022 and August 2024 (Supplementary Table S1). The study was compliant with the Declaration of Helsinki, local regulations, and Good Clinical Practice. The study was approved by the Institutional Review Board of each study site, and all patients provided written informed consent to participate. This study is registered with the Japan Registry of Clinical Trials (jRCT2031220180), and the protocol and statistical analysis plan are available at ClinicalTrials.gov (NCT05440383).

### Study population

Eligible patients were premenopausal Japanese women aged ≥20 years with uterine leiomyomas and HMB. Patients were required to have ≥1 surgically untreated and uncalcified myoma of ≥3 cm in diameter (subserosal, intramural, submucosal, and/or cervical) on transvaginal ultrasound, and a typical menstrual cycle (25–38 days) with ≥3 consecutive days of bleeding during menstruation. HMB was defined as a Pictorial Blood Loss Assessment Chart (PBAC; Higham *et al.*, 1990) total score ≥120 during the menstrual cycle preceding the treatment period.

Key exclusion criteria were: patients with a history of haematological diseases (including thalassaemia, sickle cell anaemia, folate deficiency, and coagulopathy; excluding iron deficiency anaemia and non-anaemic iron deficiency); patients with thyroid dysfunction and irregular menstruation (or those at risk of irregular menstruation due to thyroid dysfunction); patients with severe intermenstrual bleeding, significant ovulatory dysfunction-related bleeding, or abnormal genital bleeding (based on investigator judgement); patients with positive cervical cytology results ≤1 year before the start of the screening period; patients with a history of pelvic inflammatory disease or concurrent lower abdominal pain due to irritable bowel syndrome or severe interstitial cystitis (based on investigator judgement); and those with a history of osteoporosis, osteopenia, or other metabolic bone diseases. Concomitant treatment with GnRH analogues, hormonal agents, and osteoporosis medication was prohibited throughout the study. Analgesics were permitted to manage adverse events (AEs) or severe pain related to uterine leiomyomas; when used for severe leiomyoma-related pain, investigators were instructed to keep the analgesic drug and dosage as consistent as possible. Oral iron preparations were permitted in patients already receiving iron before enrolment, or in patients with blood haemoglobin <10 g/dl when enrolled.

### Study procedures

The study comprised a screening period, a run-in period, a 24-week treatment period, and a follow-up period of up to 24 weeks (Supplementary Fig. S1). During the single-blind run-in period, all patients received placebos for linzagolix and leuprorelin to minimize the placebo effect on patient-reported pain symptoms. At the start of the treatment period (Days 1–5 of the first menstruation during the run-in period), patients were randomized 1:1 to receive oral linzagolix 200 mg (two 100 mg tablets, once daily) or subcutaneous leuprorelin 1.88 mg (once every 4 weeks [Q4W]) for 24 weeks. In line with its approved posology in Japan (Pharmaceuticals and Medical Devices Agency, 2023) patients in the leuprorelin group with higher body weight or severe uterine hypertrophy (based on investigator judgement) could receive leuprorelin 3.75 mg Q4W.

Patients were randomized by an interactive web response system. In order to evaluate the effects of study treatment on leiomyoma-related pain, dynamic randomization was used to stratify patients by the presence of pain symptoms (defined as maximum numeric rating scale [NRS] score ≥4 and an NRS score ≥1 for ≥2 days during the menstrual cycle preceding the treatment period). Personnel who enrolled and assigned patients to study treatments did not have access to the random allocation sequence. To maintain blinding, patients assigned to linzagolix received a subcutaneous dose of leuprorelin placebo Q4W, and those assigned to leuprorelin administered two linzagolix placebo oral tablets, once daily for 24 weeks. Throughout the screening, run-in, and treatment periods, patients kept an electronic symptom diary in which menstrual bleeding (PBAC) and pain (NRS) were recorded each day. Patients also attended study visits at baseline and Weeks 2, 4, 8, 12, 16, 20, and 24 of the treatment period; and at Weeks 4 and 24 of the follow-up period. Study assessments performed at each visit are presented in Supplementary Table S2.

### Study outcomes

The primary endpoint was the proportion of patients with a total PBAC score <10 from Weeks 6 to 12 of the treatment period. Secondary efficacy endpoints included the proportion of patients with a total PBAC score <10 at other time points; time to a total PBAC score <10; proportion of patients with amenorrhoea (total PBAC score of 0); proportion of patients with a maximum NRS score ≤1 in those with pain symptoms at baseline; blood haemoglobin, blood haematocrit, serum iron, and serum ferritin levels; myoma and uterine volumes (measured by transvaginal ultrasound); symptom severity and HRQoL total scores on the Uterine Fibroid Symptom and Quality of Life (UFS-QOL) questionnaire (Spies *et al.*, 2002); and patient-reported symptom control based on the Patient Global Impression of Change (PGIC) scale. An additional *post hoc* efficacy endpoint was the time to a maximum NRS score ≤1 in those with pain symptoms at baseline. Pharmacodynamic endpoints included plasma oestradiol and serum LH, FSH, and progesterone levels.

Safety endpoints included AEs (coded using the Medical Dictionary for Regulatory Activities [MedDRA version 25.1]), BMD and bone density *T*-scores (measured in the lumbar spine using dual-energy X-ray absorptiometry), clinical laboratory tests, vital signs, body weight, 12-lead electrocardiogram, and the time to menstrual recovery after the last dose of study treatment. An additional *post hoc* safety endpoint was the time from the last dose of study treatment to the recovery of hot flushes.

### Statistical analysis

A sample size of 264 patients (n = 132 per treatment group) was estimated to provide 90% power to demonstrate the non-inferiority of linzagolix versus leuprorelin for the primary endpoint. Sample size calculations used a non-inferiority margin of −15% and a one-sided significance level of 2.5%, and assumed that 83% of patients in each treatment group would achieve a total PBAC score <10 from Weeks 6 to 12 (derived from previous trials of another GnRH receptor antagonist; Osuga *et al.*, 2019; Hoshiai *et al.*, 2021).

Efficacy and pharmacodynamic outcomes were assessed in the full analysis set, defined as all randomized patients who received ≥1 dose of assigned study treatment. To test the robustness of the results for the primary efficacy endpoint, supplementary analyses were performed in the per-protocol set, defined as all patients in the full analysis set who met the study eligibility criteria; remained in the study at Week 12 of the treatment period; demonstrated ≥75% compliance to study treatment and had ≥75% of daily PBAC scores recorded up to Week 12 of the treatment period; and received no prohibited concomitant treatments before Week 12 of the treatment period. Safety outcomes were assessed in the safety set, defined as all patients who received ≥1 dose of study treatment during the treatment period. Unless otherwise stated, data were summarized using descriptive statistics and, where appropriate, with between-group differences and associated 95% CIs.

The non-inferiority of linzagolix versus leuprorelin for the primary endpoint was evaluated based on the 95% CI of the between-group difference, calculated using the Farrington–Manning method. If the lower bounds of the two-sided 95% CI were equal to or greater than the non-inferiority margin (−15%), the non-inferiority of linzagolix versus leuprorelin was established.

Kaplan–Meier analyses and log-rank tests were used to evaluate the time to a total PBAC score <10, and the time to a maximum NRS score ≤1. For time to a total PBAC score <10, the total PBAC score was calculated for each successive 42 day interval (i.e. Days 1–42, 2–43, 3–44, etc.), and the event onset date was defined as the first day of the 42-day period in which a total PBAC score <10 was achieved and maintained through to the end of study treatment. For time to a maximum NRS score ≤1, the maximum NRS score was evaluated for each successive 28 day interval (i.e. Days 1–28, 2–29, 3–30, etc.), and the event onset date was defined as the first day of the 28-day period in which a maximum NRS score ≤1 was achieved and maintained through to the end of study treatment. Two-sample Wilcoxon tests were used to compare the distribution of patients across PGIC categories in each group. A two-sided significance level of 5.0% was used in statistical tests. Analyses were based on observed data with no imputation for missing values, and were performed using SAS version 9.4 (SAS Institute Inc., Cary, NC, USA).

## Results

### Study population

**Figure 1.**
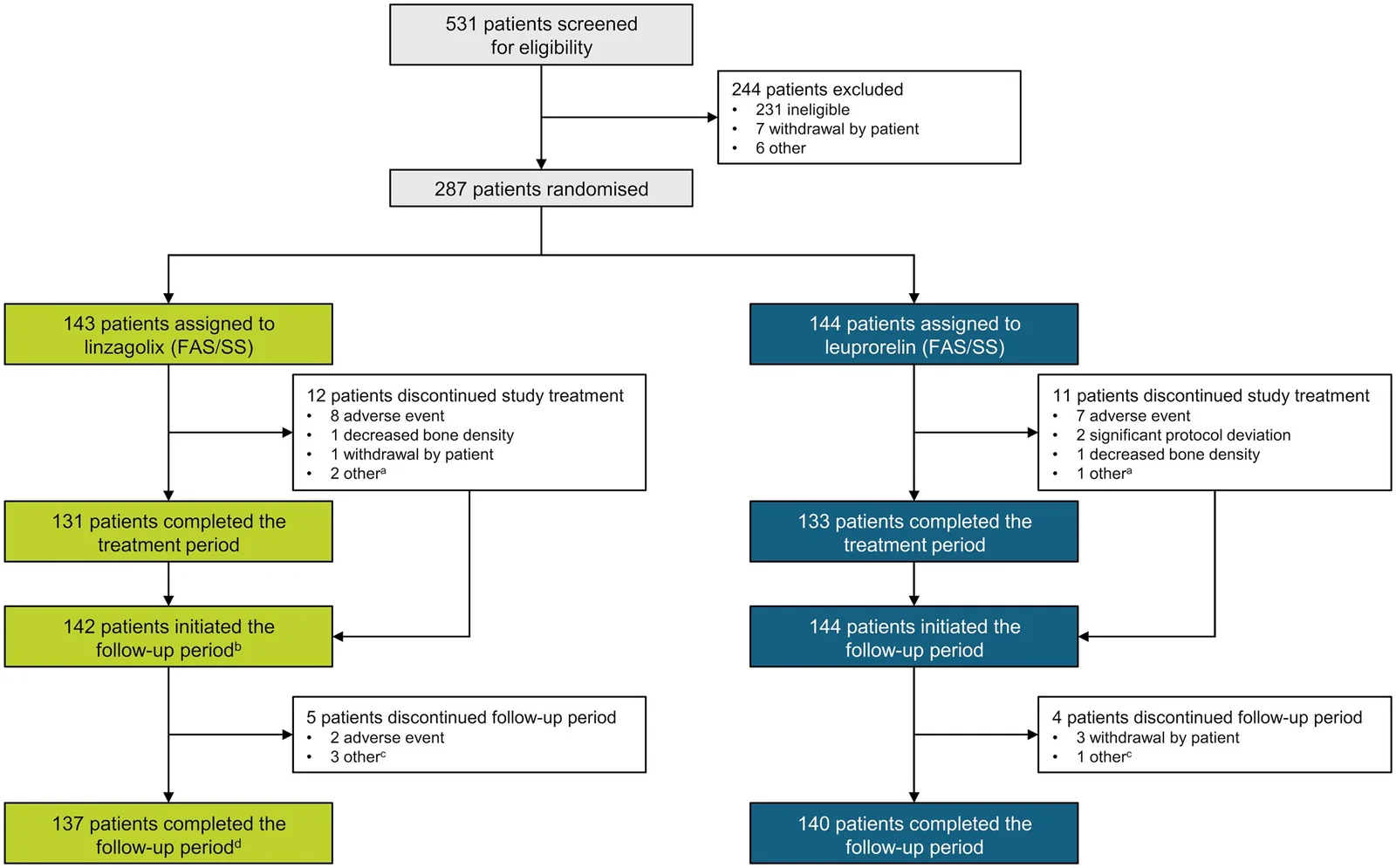
**Patient disposition**. ^a^Other reasons for discontinuing study treatment were decreased blood haemoglobin at the start of the treatment period (1 patient each in the linzagolix and leuprorelin groups) and adverse events during the screening period (1 patient in the linzagolix group). ^b^One patient who discontinued study treatment during the treatment period did not enter the follow-up period. ^c^In both groups, other reasons for discontinuing the follow-up period involved the treatment of uterine leiomyomas (including the use of prohibited concomitant treatments). ^d^Patients who discontinued study treatment before Week 12 of the treatment period were considered to have completed the follow-up period if they attended one follow-up visit 4 weeks after the last dose. FAS, full analysis set; SS, safety set.

In total, 531 patients with uterine leiomyomas and HMB were screened for eligibility (Fig. 1). After exclusions, 287 patients were enrolled and randomized to receive linzagolix (n = 143) or leuprorelin (n = 144); of these, 131 (91.6%) and 133 (92.4%), respectively, completed the treatment period. All 287 patients were included in both the full analysis and safety sets for evaluation of efficacy, pharmacodynamic, and safety outcomes. Supplementary analyses in the per-protocol set included 135 patients (94.4%) in the linzagolix group and 129 (89.6%) in the leuprorelin group.

Baseline patient characteristics were generally balanced between treatment groups (Table 1). Across groups, the mean age at baseline was 43.7 years, most patients (86.1%) had intramural uterine leiomyomas, and the mean total PBAC score was 284.7. Overall, 47.7% of patients had pain symptoms at baseline, and the mean maximum NRS score in these patients was 5.7 out of 10.

**Table 1.** Baseline patient demographics and clinical characteristics (full analysis set).

	Linzagolix (n = 143)	Leuprorelin (n = 144)	Total (N = 287)
Age (years), mean (SD)	43.7 (5.2)	43.7 (5.2)	43.7 (5.2)
BMI (kg/m^2^), mean (SD)	23.3 (3.6)	23.6 (3.8)	23.5 (3.7)
Uterine leiomyoma type, n (%)^a^			
Subserosal	59 (41.3)	50 (34.7)	109 (38.0)
Intramural	125 (87.4)	122 (84.7)	247 (86.1)
Submucosal	24 (16.8)	33 (22.9)	57 (19.9)
Cervical	3 (2.1)	3 (2.1)	6 (2.1)
Comorbidities, n (%)			
Endometriosis	12 (8.4)	14 (9.7)	26 (9.1)
Adenomyosis	10 (7.0)	13 (9.0)	23 (8.0)
Total PBAC score, mean (SD)	271.8 (154.2)	297.5 (187.2)	284.7 (171.7)
Pain symptoms, n (%)^b^	68 (47.6)	69 (47.9)	137 (47.7)
Maximum NRS score, mean (SD)^b^	5.6 (1.5)	5.8 (1.7)	5.7 (1.6)
Haemoglobin (g/dl), mean (SD)	11.9 (1.4)	11.9 (1.5)	11.9 (1.4)
<12 g/dl, n (%)	67 (46.9)	60 (41.7)	127 (44.3)
Myoma volume (cm^3^), mean (SD)	121.9 (148.6)	113.8 (140.5)	117.8 (144.4)
Uterine volume (cm^3^), mean (SD)	291.1 (234.6)	284.7 (205.2)	287.9 (220.0)
UFS-QOL score, mean (SD)^c^			
Symptom severity score	32.3 (15.0)	31.9 (15.5)	32.1 (15.2)
HRQoL total score	71.4 (19.5)	73.6 (20.1)	72.5 (19.8)
Leuprorelin dose, n (%)^d^			
1.88 mg	114 (79.7)	117 (81.3)	231 (80.5)
3.75 mg	29 (20.3)	27 (18.8)	56 (19.5)

a Patients could have multiple types of uterine leiomyomas.

b Pain symptoms at baseline was defined as a maximum NRS score ≥4 and an NRS score ≥1 for ≥2 days during the menstrual cycle preceding the treatment period; maximum NRS score was evaluated in patients with pain symptoms at baseline.

c UFS-QOL symptom severity scores range from 0 to 100, with higher scores indicating greater symptom severity; UFS-QOL HRQoL total scores range from 0 to 100, with higher scores indicating greater HRQoL.

d In the linzagolix group, the dose of leuprorelin placebo is shown.

HRQoL, health-related quality of life; NRS, numeric rating scale; PBAC, Pictorial Blood Loss Assessment Chart; UFS-QOL, Uterine Fibroid Symptom and Quality of Life.

### Efficacy outcomes

#### Menstrual bleeding

**Figure 2.**
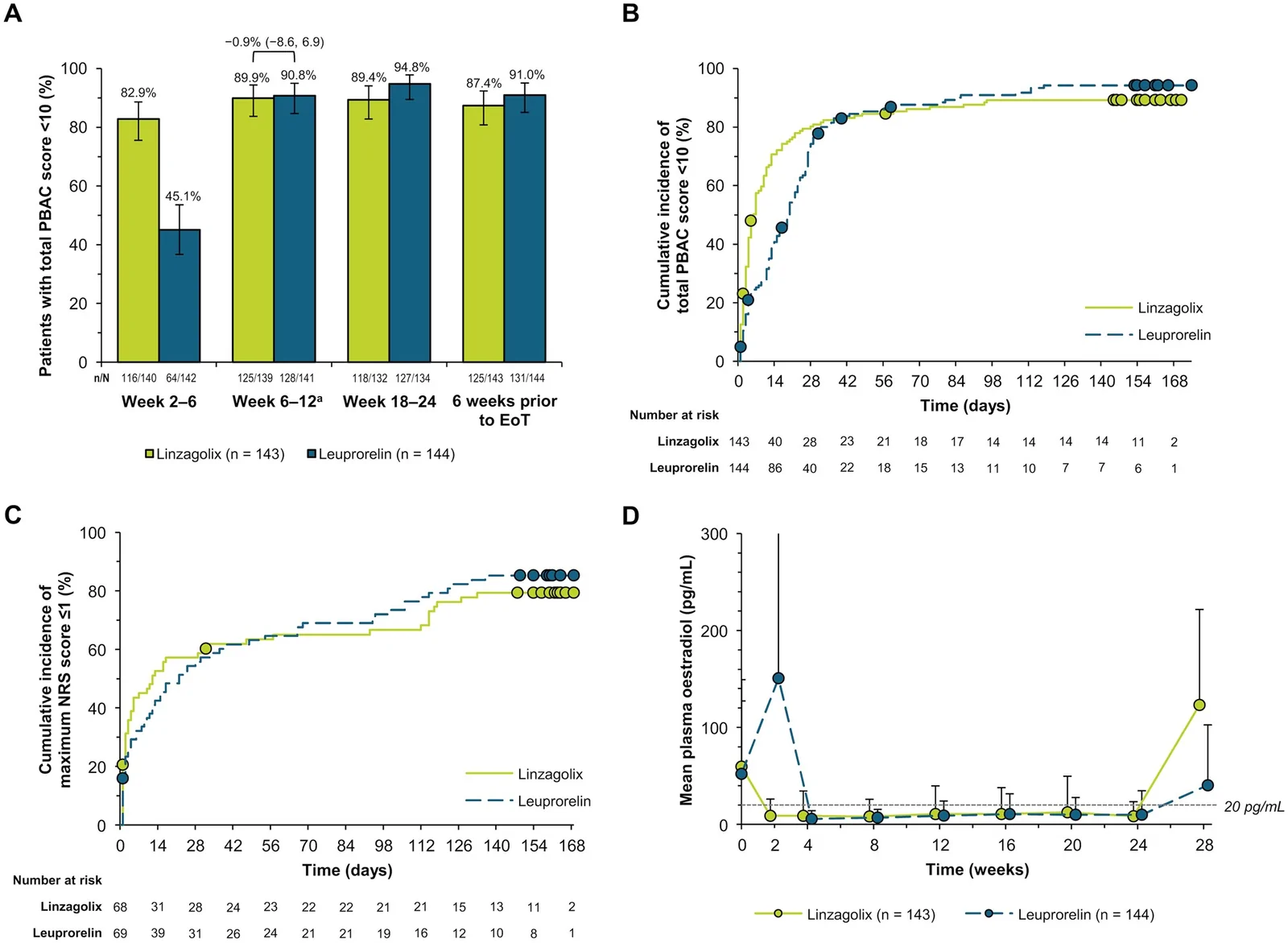
**Pictorial Blood Loss Assessment Chart (PBAC) response, pain response, and plasma oestradiol levels**. Proportion of patients with a total PBAC score <10 over time (**A**); Kaplan–Meier plots of time to a total PBAC score <10 (**B**) and time to a maximum numeric rating scale (NRS) score ≤1 (**C**); and mean plasma oestradiol levels over time (**D**). In figure A, error bars represent 95% CI; the label at Weeks 6–12 presents the between-group difference and associated 95% CI. In figure B and C, circles represent censored patients; the number at risk represents the number of patients who have not yet experienced the event or been censored at that time point. In figure B, time to a total PBAC score <10 was significantly shorter in the linzagolix group versus leuprorelin (*P *= 0.023, based on log-rank tests). In figure C, *post hoc* analyses included patients with pain symptoms at baseline. Time to a maximum NRS score ≤1 was not significantly different between the linzagolix and leuprorelin groups (*P *= 0.805, based on log-rank tests). In figure D, plasma oestradiol was measured at each visit during the treatment period and at Week 4 of the follow-up period. Error bars represent standard deviation; Week 28 represents Week 4 of the follow-up period. ^a^Primary endpoint. The proportion of patients in the linzagolix group with a total PBAC score <10 from Weeks 6 to 12 was non-inferior to that of the leuprorelin group, since the lower bounds of the 95% CI for the between-group difference were greater than the prespecified non-inferiority margin of −15%. EoT, end of treatment.

In the primary endpoint analysis, the proportion of patients with a total PBAC score <10 from Weeks 6 to 12 was 89.9% (95% CI, 83.7, 94.4) in the linzagolix group and 90.8% (84.7, 95.0) in the leuprorelin group, yielding a between-group difference of −0.9% (−8.6, 6.9; Fig. 2A). Since the lower bounds of the 95% CI for the between-group difference was greater than the prespecified non-inferiority margin of −15%, the non-inferiority of linzagolix versus leuprorelin was established. In supplementary analyses, non-inferiority for the primary endpoint was similarly achieved in the per-protocol set, thus confirming the robustness of the primary analysis results (Supplementary Table S3). In other assessment periods, the proportion of patients with a total PBAC score <10 from Weeks 2 to 6 was 82.9% in the linzagolix group versus 45.1% in the leuprorelin group, indicating rapid improvements in HMB with linzagolix (Fig. 2A). In subgroup analyses, the proportion of patients with a total PBAC score <10 from Weeks 6 to 12 was similarly high and comparable between groups in patients stratified by key baseline factors (Supplementary Table S4).

The time to a total PBAC score <10 was significantly shorter among patients receiving linzagolix versus leuprorelin (*P *= 0.023; Fig. 2B). The median time to a total PBAC score <10 was 6.0 days (95% CI, 4.0, 8.0) in the linzagolix group, versus 20.0 days (15.0, 23.0) in the leuprorelin group. The proportion of patients with amenorrhoea (total PBAC score of 0) was greater for the linzagolix group from Weeks 2 to 6 (65.0% vs 26.8% in the leuprorelin group), and similar between groups thereafter (Table 2).

**Table 2.** Summary of secondary efficacy outcomes (full analysis set).

	Linzagolix (n = 143)	Leuprorelin (n = 144)	Difference vs leuprorelin (95% CI)
Patients with amenorrhoea (total PBAC score of 0), n/N (%)			
Weeks 2–6	91/140 (65.0)	38/142 (26.8)	38.2 (26.7, 49.7)
Weeks 6–12	110/139 (79.1)	116/141 (82.3)	−3.1 (−12.5, 6.2)
Weeks 18–24	117/132 (88.6)	120/134 (89.6)	−0.9 (−9.1, 7.3)
Patients with a maximum NRS score ≤1, n/N (%)^a^			
28 days prior to Week 12^b^	49/68 (72.1)	55/69 (79.7)	−7.7 (−21.9, 6.6)
28 days prior to EoT	53/68 (77.9)	58/69 (84.1)	−6.1 (−19.4, 7.1)
Haemoglobin (g/dl), mean (SD)^c^			
Week 12	13.0 (1.1)	13.1 (1.1)	−0.1 (−0.3, 0.2)
Week 24	13.2 (1.0)	13.3 (0.9)	−0.1 (−0.3, 0.1)
Patients with haemoglobin ≥12 g/dl, n/N (%)^c^^,^^d^			
Week 12	39/62 (62.9)	37/55 (67.3)	−4.4 (−21.4, 12.7)
Week 24	54/61 (88.5)	44/53 (83.0)	5.5 (−8.0, 19.0)
Percent change in myoma volume from baseline (%), mean (SD)			
Week 2	−17.9 (40.7)	−5.2 (40.9)	−12.7 (−22.4, −3.1)
Week 12	−47.7 (26.6)	−41.7 (31.7)	−5.9 (−12.9, 1.1)
Week 24	−53.3 (30.7)	−50.8 (28.5)	−2.5 (−9.7, 4.7)
Percent change in uterine volume from baseline (%), mean (SD)			
Week 2	−23.0 (19.1)	−5.0 (32.6)	−18.0 (−24.3, −11.7)
Week 12	−41.9 (22.0)	−39.8 (27.7)	−2.0 (−8.0, 3.9)
Week 24	−45.6 (26.6)	−43.7 (31.8)	−1.9 (−9.0, 5.2)
Change in UFS-QOL symptom severity score from baseline, mean (SD)^e^			
Week 12	−24.9 (15.3)	−25.3 (15.0)	0.4 (−3.2, 4.0)
Week 24	−24.6 (15.6)	−26.1 (14.6)	1.5 (−2.2, 5.1)
Change in UFS-QOL HRQoL total score from baseline, mean (SD)^e^			
Week 12	17.0 (20.0)	16.8 (18.7)	0.3 (−4.4, 4.9)
Week 24	18.5 (20.4)	17.5 (18.9)	1.0 (−3.8, 5.8)
Patients with PGIC of ‘very much improved’ or ‘much improved’, n/N (%)^c^^,^^f^			
Week 12	95/136 (69.9)	88/137 (64.2)	5.6 (−5.5, 16.7)
Week 24	102/132 (77.3)	91/131 (69.5)	7.8 (−2.9, 18.6)

a Analysis included patients with pain symptoms at baseline, which was defined as a maximum NRS score ≥4 and an NRS score ≥1 for ≥2 days during the menstrual cycle preceding the treatment period.

b Defined as Days 57–84 for patients who reached Week 12, and 28 days prior to EoT for patients who discontinued prior to Week 12.

c Between-group differences were evaluated in *post hoc* analyses.

d Analysis included patients with haemoglobin <12 g/dl at baseline.

e Reductions in UFS-QOL symptom severity scores from baseline indicate improvements in symptom severity; increases in UFS-QOL HRQoL total scores from baseline indicate improvements in HRQoL.

f Patients rated their overall symptoms due to uterine leiomyomas as ‘very much improved’, ‘much improved’, minimally improved’, ‘no change’, ‘minimally worse’, ‘much worse’, or ‘very much worse’, relative to their overall symptoms before the start of the run-in period.

EoT, end of treatment; HRQoL, health-related quality of life; NRS, numeric rating scale; PBAC, Pictorial Blood Loss Assessment Chart; PGIC, Patient Global Impression of Change; SD, standard deviation; UFS-QOL, Uterine Fibroid Symptom and Quality of Life.

#### Pain

Among those with pain symptoms at baseline, the proportion of patients with a maximum NRS score ≤1 improved over time and was generally comparable between treatment groups (Table 2 and Supplementary Table S5). In the 28 days prior to Week 12, 72.1% of patients in the linzagolix group achieved a maximum NRS score ≤1, versus 79.7% of those in the leuprorelin group. Although the time to a maximum NRS score ≤1 was not significantly different between groups in *post hoc* Kaplan–Meier analyses (*P *= 0.805), the median time to a maximum NRS score ≤1 was numerically shorter in the linzagolix group (12.0 days [95% CI, 4.0, 33.0]) versus leuprorelin (22.0 days [11.0, 39.0]; Fig. 2C).

#### Anaemia

Improvements in blood haemoglobin, blood haematocrit, serum iron, and serum ferritin levels were comparable between groups (Table 2 and Supplementary Table S6). Among those with anaemia (haemoglobin <12 g/dl) at baseline, 88.5% of patients in the linzagolix group and 83.0% in the leuprorelin group achieved blood haemoglobin ≥12 g/dl at Week 24. Similar results were observed in the subgroup of patients receiving oral iron supplementation for anaemia.

#### Myoma and uterine volumes

Reductions in myoma and uterine volumes were more rapid in the linzagolix group versus leuprorelin (Table 2). The mean percent change in myoma volume from baseline at Week 2 was −17.9% in the linzagolix group versus −5.2% in the leuprorelin group, which improved to comparable levels at Week 12 (−47.7% vs −41.7%, respectively) and then was maintained at Week 24 (−53.3% vs −50.8%). Similarly, the mean percent reduction in uterine volume from baseline was greater in the linzagolix group at Week 2 (−23.0% vs −5.0% in the leuprorelin group), then was comparable between groups at Week 12 (−41.9% vs −39.8%) and Week 24 (−45.6% vs −43.7%).

#### Patient-reported outcomes

Improvements in UFS-QOL symptom severity and HRQoL total scores were comparable between groups (Table 2). Similarly, the distribution of patients across PGIC categories was not significantly different between groups at Week 12 (*P *= 0.547) or Week 24 (*P *= 0.513; Supplementary Table S7). The proportion of patients whose symptoms were ‘very much improved’ or ‘much improved’ according to the PGIC was 77.3% in the linzagolix group at Week 24, versus 69.5% in the leuprorelin group (Table 2).

### Pharmacodynamic outcomes

Plasma oestradiol levels in the linzagolix group were rapidly reduced by direct GnRH receptor inhibition, maintained at menopausal levels (<20 pg/ml) throughout the treatment period, and recovered earlier than in the leuprorelin group after treatment cessation (Fig. 2D and Supplementary Table S8). Similar results were observed for serum LH, FSH, and progesterone levels. The proportion of patients with plasma oestradiol <20 pg/ml at Week 2 was 89.9% in the linzagolix group versus 63.6% in the leuprorelin group.

### Safety outcomes

#### Adverse events

AEs were reported in 139 patients in each of the linzagolix (97.2%) and leuprorelin (96.5%) groups (Table 3). AEs that occurred in ≥10% of patients in either treatment group were hot flush, breakthrough bleeding, nasopharyngitis, hyperhidrosis, arthralgia, and headache. Treatment-related AEs that occurred in ≥10% of patients in either group were hot flush, breakthrough bleeding, and hyperhidrosis.

**Table 3.** Summary of adverse events during the treatment period (safety set).

Patients with ≥1 AE, n (%)	Linzagolix (n = 143)	Leuprorelin (n = 144)
Any AE	139 (97.2)	139 (96.5)
Mild	121 (84.6)	114 (79.2)
Moderate	17 (11.9)	24 (16.7)
Severe	1 (0.7)	1 (0.7)
Treatment-related AEs	120 (83.9)	131 (91.0)
AEs leading to treatment interruption	3 (2.1)	0
AEs leading to treatment discontinuation	8 (5.6)	7 (4.9)
Serious AEs	2 (1.4)	2 (1.4)
AEs leading to death	0	0
AEs occurring in ≥10% of patients		
Hot flush	77 (53.8)	77 (53.5)
Breakthrough bleeding	57 (39.9)	80 (55.6)
Nasopharyngitis	33 (23.1)	29 (20.1)
Hyperhidrosis	19 (13.3)	17 (11.8)
Arthralgia	18 (12.6)	14 (9.7)
Headache	15 (10.5)	15 (10.4)

For any AEs by severity, patients with multiple AEs were counted only once, at the maximum severity.

AE, adverse event.

Most AEs were mild in severity; severe AEs occurred in one patient (0.7%) in each treatment group (Table 3). In both groups, the incidence of AEs was highest during the first 28 days of the treatment period. During this period, breakthrough bleeding was the most common AE in the linzagolix and leuprorelin groups, reported in 32.9% and 51.4% of patients, respectively.

The incidence of AEs leading to treatment discontinuation was 5.6% in the linzagolix group and 4.9% in the leuprorelin group (Table 3). Serious AEs were reported in two patients in each treatment group, including subarachnoid haemorrhage and abdominal pain in the linzagolix group (1 patient each), and vestibular neuronitis and heavy genital bleeding in the leuprorelin group (1 patient each). In particular, subarachnoid haemorrhage occurred on Day 37 of the treatment period, led to linzagolix discontinuation, and resolved without sequelae. This event was deemed to be a treatment-related serious AE by the investigator, since no other causative factors could be identified and a causal relationship could not be ruled out.

Among those with hot flushes (the most common AE overall) at the end of the treatment period, a *post hoc* analysis found that the median time from the last dose of study treatment to the recovery of hot flushes was 21 days (interquartile range [IQR], 11–44) in the linzagolix group (n = 64) versus 83 days (51–105) in the leuprorelin group (n = 58).

#### Bone mineral density

Reductions in BMD were observed in both groups over the treatment period (Supplementary Fig. S2). In the linzagolix group, the mean percent change in BMD from baseline was −2.6% at Week 12 and −5.3% at Week 24, versus −1.7% and −4.2%, respectively, in the leuprorelin group. BMD tended to increase after linzagolix cessation; at Week 24 of the follow-up period, the mean percent change in BMD from baseline was −2.8% in the linzagolix group versus −3.7% in patients who received leuprorelin.

Most patients maintained their baseline bone density *T*-score category during the treatment and follow-up periods (Supplementary Table S9). One patient in the linzagolix group with a *T*-score of >−2.5 and ≤−1 (indicating osteopenia) at baseline experienced a *T*-score reduction to ≤−2.5 (indicating osteoporosis) at Week 24 of the treatment period and at Week 24 of the follow-up period. In this patient, *T*-scores at these time points were −2.4, −2.8, and −2.6, respectively, suggesting small changes in *T*-scores overall and a trend towards recovery after the end of treatment.

#### Other safety outcomes

In both groups, small increases in liver function tests (aspartate transaminase, alanine transaminase, γ-glutamyl transferase) and blood lipids (high- and low-density lipoprotein cholesterol, total cholesterol, triglycerides) were observed during the treatment period. These increases were not clinically significant and generally reversible after treatment cessation, and none were associated with elevations in bilirubin, international normalized ratio, or alkaline phosphatase. There were no clinically significant findings in any other laboratory parameters, nor in assessments of vital signs, body weight, or 12-lead electrocardiogram parameters.

#### Time to menstrual recovery

Time to menstrual recovery in the linzagolix group was almost three times shorter than that observed in the leuprorelin group. The median time from the last dose of study treatment to menstruation was 30 days (IQR, 27–32) in patients receiving linzagolix, versus 89 days (79–106) in those receiving leuprorelin.

## Discussion

In this phase 3 study involving Japanese women with uterine leiomyomas and HMB, linzagolix met its primary endpoint and offered reductions in menstrual bleeding that were non-inferior to leuprorelin from Weeks 6 to 12 of the treatment period. Improvements in pain, anaemia-related outcomes, myoma and uterine volumes, UFS-QOL scores, and PGIC were also achieved with linzagolix treatment, and were comparable with that observed in the leuprorelin group by Week 24. These clinical benefits were consistent with those reported in the phase 3 PRIMROSE 1 and 2 trials conducted in the USA and Europe (Donnez *et al.*, 2022, 2025), thereby confirming the efficacy of linzagolix in a Japanese patient population. Linzagolix also demonstrated a favourable safety profile; most AEs were mild in severity, and safety outcomes were generally comparable between treatment groups. Overall, these data support the use of linzagolix over 24 weeks for the treatment of Japanese patients with symptomatic uterine leiomyomas.

Clinical improvements achieved with linzagolix in the present study (including HMB, pain, haemoglobin levels, myoma/uterine volumes, and HRQoL) were in line with previous placebo-controlled trials of other GnRH receptor antagonists (e.g. relugolix, elagolix) (Schlaff *et al.*, 2020; Al-Hendy *et al.*, 2021), and in particular, a phase 3 study of relugolix versus leuprorelin in Japanese women with symptomatic uterine leiomyomas (Osuga *et al.*, 2019). This previous study similarly demonstrated the non-inferiority of relugolix versus leuprorelin for the proportion of patients with a total PBAC score <10 from Weeks 6 to 12; comparable improvements in pain, haemoglobin, and UFS-QOL scores between groups; and earlier reductions in menstrual bleeding and myoma/uterine volumes with relugolix due to rapid reductions in oestradiol levels with no flare-up effect (Osuga *et al.*, 2019). Improvements in HMB with relugolix were observed as early as Weeks 2–6 of the study and reductions in pain were observed by Weeks 6–12 (Osuga *et al.*, 2019); in comparison, Kaplan–Meier analyses in our study estimated that the median time to a total PBAC score <10 was 6 days with linzagolix (vs 20 days with leuprorelin), and the median time to a maximum NRS score of ≤1 was 12 days (vs 22 days). Rapid reductions in HMB were also observed in PRIMROSE 1 and 2, where it was estimated that the median time to a clinically significant HMB reduction was only 3 days for linzagolix 200 mg (Donnez *et al.*, 2025). Considering the burden that HMB and pain impose on daily activities and HRQoL (Fortin *et al.*, 2018; Go *et al.*, 2020), linzagolix and its rapid onset of action may represent a favourable treatment option for symptomatic uterine leiomyomas.

In addition to its earlier onset of action, the present study also found that linzagolix was associated with an earlier recovery of plasma oestradiol levels after the end of treatment. Similar to the previous phase 3 study of relugolix (Osuga *et al.*, 2019), this finding coincided with an earlier resumption of menses in the linzagolix group versus leuprorelin. The new insight gained from this study was that we also observed an earlier recovery of safety outcomes related to hypo-oestrogenism, namely hot flushes and reductions in BMD. These results support the notion that pituitary recovery after discontinuation of a GnRH receptor antagonist is faster than that of a GnRH agonist (Ciebiera *et al.*, 2023), which may be an important consideration for women planning pregnancy after treatment or those experiencing hypo-oestrogenic side effects.

Overall, linzagolix demonstrated a favourable safety profile that was comparable with leuprorelin in this study, and generally consistent with previous reports of relugolix and elagolix in women with uterine leiomyomas and HMB (Osuga *et al.*, 2019; Schlaff *et al.*, 2020; Al-Hendy *et al.*, 2021). Similar to PRIMROSE 1 and 2 (Donnez *et al.*, 2022), the most common AE reported among the linzagolix group in this study was hot flush (53.8%), and this reflects the hypo-oestrogenic state induced by GnRH receptor antagonists. The incidence of breakthrough bleeding was lower in the linzagolix group versus leuprorelin, due to the absence of the initial flare-up effect. Loss of BMD is another menopause-like symptom of GnRH analogue treatment, and while this class effect was observed in both groups, no clinically significant changes in *T*-scores were reported. There were also no clinically significant changes in any other safety outcomes evaluated in this study, further supporting the safety of linzagolix for the management of uterine leiomyomas and HMB.

To our knowledge, this study was the first active comparator-controlled trial to evaluate the efficacy, safety, and pharmacodynamic effects of linzagolix in Japanese women with symptomatic uterine leiomyomas. While these were demonstrated in a diverse group that included patients with different leiomyoma types, comorbid endometriosis, and/or adenomyosis, we acknowledge that further studies are needed to examine the influence of detailed uterine leiomyoma phenotypes—such as those based on International Federation of Obstetrics and Gynaecology (FIGO) classifications (Munro *et al.*, 2018)—on treatment outcomes. Moreover, patients in this study were selected based on the presence of uterine leiomyomas on transvaginal ultrasound and menstrual bleeding assessed by PBAC; however, investigations to further characterize leiomyomas (e.g. magnetic resonance imaging) and other potential causes of HMB (e.g. adenomyosis, primary endometrial disorders, coagulopathies) were not performed. Despite the chronic nature of uterine leiomyomas and the current unmet need for longer-term treatment options, this study was also limited to a relatively short treatment period of 24 weeks, due to the potential risk of BMD loss with linzagolix. Although the menopause-like effects of linzagolix may present a barrier to long-term treatment, data from PRIMROSE 1 and 2 suggest that this may be overcome with hormonal add-back therapy or with a partial suppression dose (100 mg) of linzagolix (Donnez *et al.*, 2022). Therefore, further studies are warranted to determine whether linzagolix, with or without hormonal add-back therapy, may be a safe and efficacious long-term treatment option for Japanese patients with symptomatic uterine leiomyomas.

## Conclusions

This phase 3 study demonstrated the efficacy and safety of linzagolix, an oral GnRH receptor antagonist, in Japanese women with uterine leiomyomas and HMB. Once-daily treatment with linzagolix was associated with improvements in HMB, pain, anaemia, myoma and uterine volumes, patient-reported symptom severity, and HRQoL that were generally comparable with leuprorelin over 24 weeks. However, linzagolix also demonstrated an earlier onset of action for several key endpoints (e.g. HMB, pain, myoma/uterine volumes), and an earlier resolution of others after treatment cessation (e.g. plasma oestradiol levels, menstrual recovery), highlighting potential advantages of GnRH receptor inhibition over existing GnRH agonists. Together, these findings support the use of linzagolix over 24 weeks to manage the signs and symptoms of uterine leiomyomas.

## Supplementary Material

deag106_Supplementary_Figure_S1

deag106_Supplementary_Figure_S2

deag106_Supplementary_Table_S1

deag106_Supplementary_Table_S2

deag106_Supplementary_Table_S3

deag106_Supplementary_Table_S4

deag106_Supplementary_Table_S5

deag106_Supplementary_Table_S6

deag106_Supplementary_Table_S7

deag106_Supplementary_Table_S8

deag106_Supplementary_Table_S9

## Data Availability

Individual Patient Data (IPD) are available upon reasonable request and with the permission of Kissei Pharmaceutical Co., Ltd. For requests to access the IPD, please contact the corresponding author.
