# Supplementary material for: Linzagolix versus leuprorelin in Japanese women with uterine leiomyomas: a phase 3, randomized, active-controlled, non-inferiority trial
Source: Hum Reprod. 2026 Jul 6;41(9):1534–43. doi: 10.1093/humrep/deag106 (PMC13550677; doi:10.1093/humrep/deag106)
Supplement: Supplementary file 1 [file deag106_supplementary_figure_s1.pdf]

Human Reproduction, 2026, 1–1
https://doi.org/10.1093/humrep/deag106
Supplementary Data

human reproduction
OXFORD

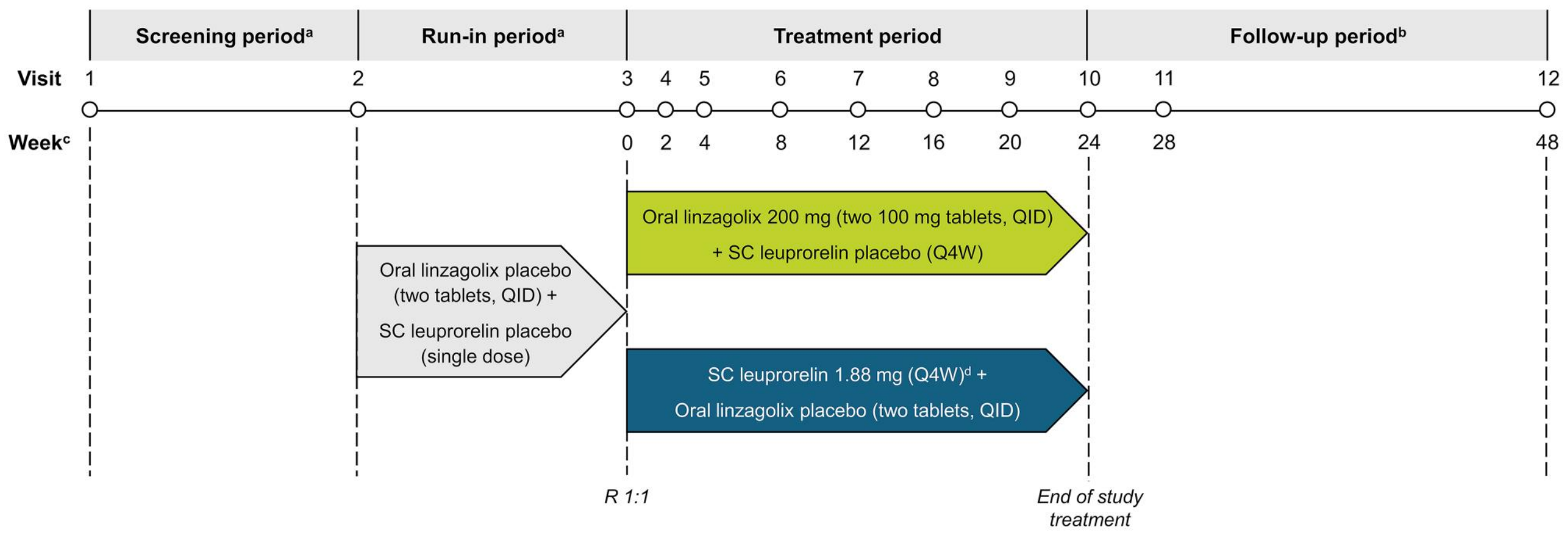

Supplementary Figure S1. Study design. [a]The screening period was defined as the time between the screening visit and the next visit during Days 1–5 of the first menstruation after screening (visit 2); the run-in period was defined as the time between visit 2 and the next visit during Days 1–5 of the second menstruation after screening (visit 3). [b]For patients who discontinued study treatment before Week 24 of the treatment period, visits during the follow-up period occurred 4 and 24 weeks after the last dose. For those who discontinued study treatment before Week 12, only one follow-up visit 4 weeks after the last dose was required. [c]Weeks after the start of the treatment period. [d]In line with its approved posology in Japan, patients in the leuprorelin group with higher body weight or severe uterine hypertrophy (based on investigator judgement) could receive SC leuprorelin 3.75 mg Q4W. QID, once daily; Q4W, once every 4 weeks; R, randomization; SC, subcutaneous.
