# Supplementary material for: Linzagolix versus leuprorelin in Japanese women with uterine leiomyomas: a phase 3, randomized, active-controlled, non-inferiority trial
Source: Hum Reprod. 2026 Jul 6;41(9):1534–43. doi: 10.1093/humrep/deag106 (PMC13550677; doi:10.1093/humrep/deag106)
Supplement: Supplementary file 2 [file deag106_supplementary_figure_s2.pdf]

Human Reproduction, 2026, 1–1
https://doi.org/10.1093/humrep/deag106
Supplementary Data

human reproduction
OXFORD

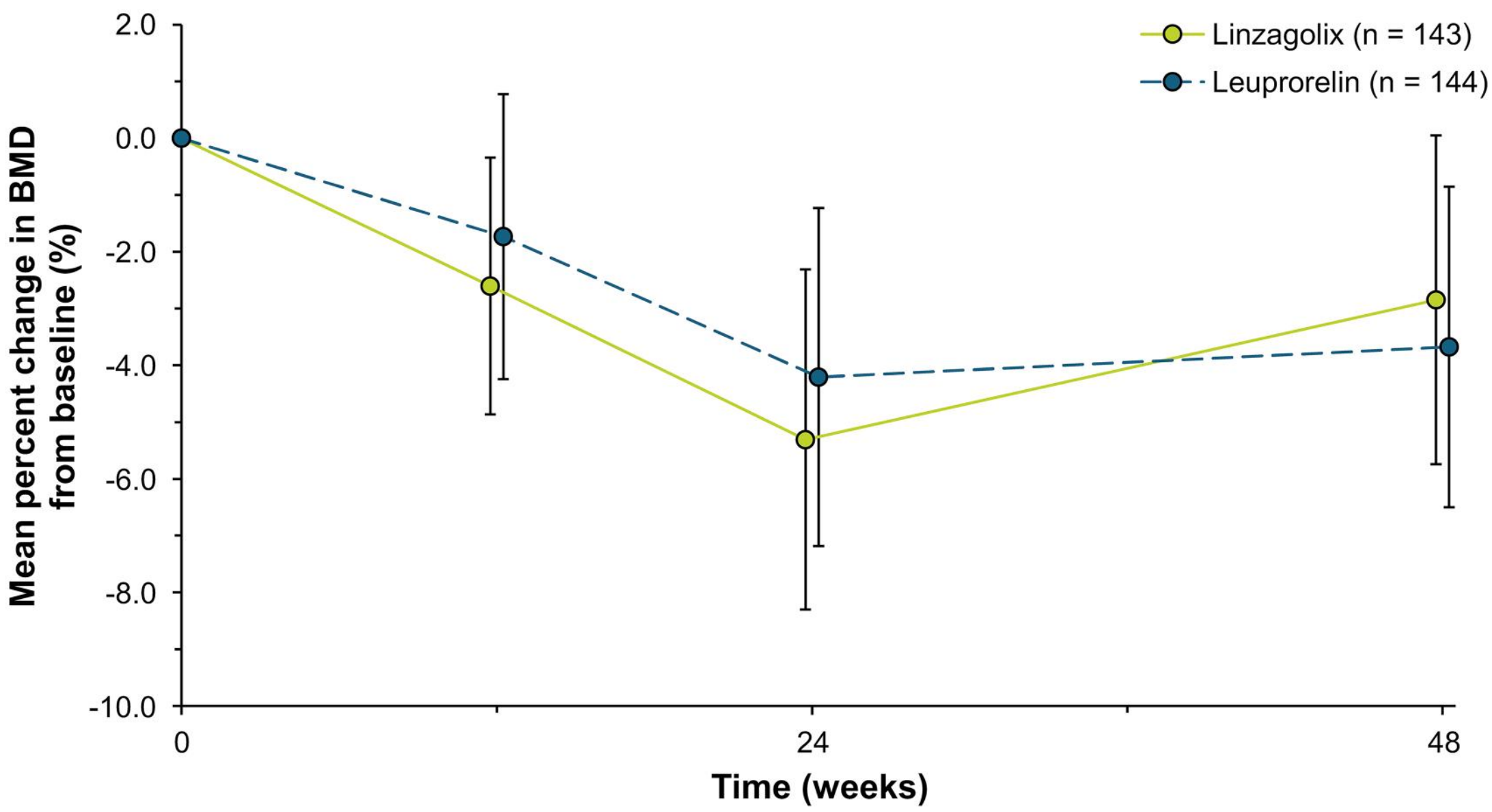

Supplementary Figure S2. **Mean percent change in BMD from baseline over time (safety set).** Weeks 0–24 represent the treatment period; Weeks 24–48 represent the follow-up period. Error bars represent standard deviation. BMD, bone mineral density.
