# Supplementary material for: Linzagolix versus leuprorelin in Japanese women with uterine leiomyomas: a phase 3, randomized, active-controlled, non-inferiority trial
Source: Hum Reprod. 2026 Jul 6;41(9):1534–43. doi: 10.1093/humrep/deag106 (PMC13550677; doi:10.1093/humrep/deag106)
Supplement: Supplementary file 3 [file deag106_supplementary_table_s1.pdf]

Human Reproduction, 2026, 1–1
https://doi.org/10.1093/humrep/deag106
Supplementary Data

human reproduction

OXFORD

Supplementary Table S1. KLH2301 study investigators.

Investigator name[a]	Study site
Futoshi Arakane	Japanese Red Cross Kumamoto Hospital, Kumamoto
Masao Fukuhara	Fukuoka Sanno Hospital, Fukuoka
Hideki Hanashi	New Medical Research System Clinic, Tokyo
Akitoshi Hasegawa	Second Kawasaki Saiwai Clinic, Kanagawa
Masaki Hashimoto	Hashimoto Clinic, Hokkaido
Mariko Hatta	Iryohojinshadan Seijunkai Juno Vesta Clinic Hatta, Chiba
Jun Hayakawa	Hayakawa Clinic, Osaka
Kozo Hirai	GyNet Medical Corporation Minamimorimachi Ladies' Clinic, Osaka
Nobuyuki Hosoi	MEITETSU Hospital, Aichi
Shinya Iwasaki	Shizuoka City Shimizu Hospital, Shizuoka
Takayuki Kamiya	Tokyo Asbo Clinic, Tokyo
Yuji Kashiwazaki	KASHIWAZAKI OB/GYN CLINIC, Saitama
Fuminori Kitada	Suita Tokushukai Hospital, Osaka
Takashi Kitanaka	KITANAKA Ladies Clinic, Osaka
Masanori Maruyama	Maruyama Memorial General Hospital, Saitama
Ryuji Nagai	Kumamoto Shinto General Hospital, Kumamoto
Sonoe Nishiguchi	Asahi-Clinic, Kagawa
Akira Nishikawa	NISHIKAWA Women's Health Clinic, Hokkaido
Shunji Nojima	National Hospital Organization Kanazawa Medical Center, Ishikawa
Akinori Oki	Ibaraki Prefectural Central Hospital, Ibaraki
Hisato Oku	Women's Clinic WE Osaka, Osaka
Masatoshi Sano	SANO Women's Clinic, Fukuoka
Mari Sawada	Kurashiki Medical Clinic, Okayama
Yukari Sumi	TORANOMON WOMENS CLINIC, Tokyo
Akiko Tanabe	jMOG Medical Corporation Tanabe Ladies' Clinic, Osaka
Chisei Tei	Sei Women's Clinic, Tokyo
Mizue Teramoto	NTT Medical Center Sapporo, Hokkaido
Ruriko Tsushima	Tsushima Ruriko Women's Life Clinic Ginza, Tokyo
Rikako Uchida	Rikako Ladies Clinic, Osaka
Kenji Umayahara	KYOUNDO HOSPITAL, Tokyo
Mamoru Urabe	Omi Medical Center, Shiga
Yukari Utsugisawa	Women's Clinic LUNA Yokohama Motomachi, Kanagawa
Akiyoshi Yamanaka	Chayamachi Ladies Clinic, Osaka
Teruko Yasuda	Yoshio Clinic, Hokkaido
Shohei Yoshida	Ginza Yoshida Clinic, Tokyo
Masuo Yoshioka	SUZURAN CLINIC, Tokyo
Sumie Yukawa	Yukawa Women's Clinic, Tokyo

[a] One investigator did not give consent to be named in this publication.
