# Supplementary material for: Linzagolix versus leuprorelin in Japanese women with uterine leiomyomas: a phase 3, randomized, active-controlled, non-inferiority trial
Source: Hum Reprod. 2026 Jul 6;41(9):1534–43. doi: 10.1093/humrep/deag106 (PMC13550677; doi:10.1093/humrep/deag106)
Supplement: Supplementary file 4 [file deag106_supplementary_table_s2.pdf]

Human Reproduction, 2026, 1–1
https://doi.org/10.1093/humrep/deag106
Supplementary Data

human reproduction
OXFORD

Supplementary Table S2. Study assessments.

			Treatment period								Follow-up period[b]	
	Screening period[a]	Run-in period[a]	Baseline	Week 2	Week 4	Week 8	Week 12	Week 16	Week 20	Week 24	Week 28	Week 48
Visit	1	2	3	4	5	6	7	8	9	10	11	12
Patient characteristics	X	X	X									
Symptom diary (PBAC, NRS)	→	→	→	→	→	→	→	→	→	→		
Clinical laboratory tests[c]	X	X	X		X	X	X	X	X	X	X	
Myoma and uterine volumes[d]	X		X	X	X	X	X			X		
UFS-QOL			X		X	X	X			X		
PGIC					X	X	X			X		
Pharmacodynamic tests[e]			X	X	X	X	X	X	X	X	X	
Adverse events	→	→	→	→	→	→	→	→	→	→	→	→
Bone density[f]		X					X			X		X
Vital signs	X	X	X		X	X	X	X	X	X	X	
Body weight	X	X	X		X	X	X	X	X	X	X	
12-lead electrocardiogram	X	X	X	X	X		X			X	X	
Menstrual recovery											X	X

[a] The screening period was defined as the time between the screening visit and the next visit during Days 1–5 of the first menstruation after screening (visit 2); the run-in period was defined as the time between visit 2 and the next visit during Days 1–5 of the second menstruation after screening (visit 3).

[b] For patients who discontinued study treatment before Week 24 of the treatment period, visits during the follow-up period occurred 4 and 24 weeks after the last dose. For those who discontinued study treatment before Week 12, only one follow-up visit 4 weeks after the last dose was required.

[c] Clinical laboratory tests included haematology, blood biochemistry, and urinalysis.

[d] Myoma and uterine volumes were measured by transvaginal ultrasound.

[e] Pharmacodynamic tests included plasma oestradiol, serum LH, serum FSH, and serum progesterone.

[f] Bone mineral density and bone density *T*-scores were measured in the lumbar spine using dual-energy X-ray absorptiometry.

NRS, numeric rating scale; PBAC, Pictorial Blood Loss Assessment Chart; PGIC, Patient Global Impression of Change; UFS-QOL, Uterine Fibroid Symptom and Quality of Life.
