# Supplementary material for: Linzagolix versus leuprorelin in Japanese women with uterine leiomyomas: a phase 3, randomized, active-controlled, non-inferiority trial
Source: Hum Reprod. 2026 Jul 6;41(9):1534–43. doi: 10.1093/humrep/deag106 (PMC13550677; doi:10.1093/humrep/deag106)
Supplement: Supplementary file 5 [file deag106_supplementary_table_s3.pdf]

Human Reproduction, 2026, 1–1
https://doi.org/10.1093/humrep/deag106
Supplementary Data

human reproduction

OXFORD

Supplementary Table S3. Proportion of patients with a total PBAC score <10 from Weeks 6 to 12 (per-protocol set).

n (%) (95% CI)	Linzagolix (n = 135)	Leuprorelin (n = 129)	Difference vs leuprorelin (95% CI)
Weeks 6–12	123 (91.1)<br>(85.0, 95.3)	118 (91.5)<br>(85.3, 95.7)	−0.4 (−8.1, 7.4)

The proportion of patients in the linzagolix group with a total PBAC score <10 from Weeks 6 to 12 was non-inferior to that of the leuprorelin group, since the lower bounds of the 95% CI for the between-group difference was greater than the prespecified non-inferiority margin of −15%.
PBAC, Pictorial Blood Loss Assessment Chart.
