# Supplementary material for: Linzagolix versus leuprorelin in Japanese women with uterine leiomyomas: a phase 3, randomized, active-controlled, non-inferiority trial
Source: Hum Reprod. 2026 Jul 6;41(9):1534–43. doi: 10.1093/humrep/deag106 (PMC13550677; doi:10.1093/humrep/deag106)
Supplement: Supplementary file 6 [file deag106_supplementary_table_s4.pdf]

Human Reproduction, 2026, 1–1
https://doi.org/10.1093/humrep/deag106
Supplementary Data

human reproduction

OXFORD

Supplementary Table S4. Proportion of patients with a total PBAC score <10 from Weeks 6 to 12 in subgroups stratified by baseline factors (full analysis set).

n/N (%) (95% CI)	Linzagolix (n = 143)	Leuprorelin (n = 144)
Age (years)		
<40	20/22 (90.9)<br>(70.8, 98.9)	22/24 (91.7)<br>(73.0, 99.0)
≥40	105/117 (89.7)<br>(82.8, 94.6)	106/117 (90.6)<br>(83.8, 95.2)
BMI (kg/m^2)		
<25	91/101 (90.1)<br>(82.5, 95.1)	89/96 (92.7)<br>(85.6, 97.0)
≥25	34/38 (89.5)<br>(75.2, 97.1)	39/45 (86.7)<br>(73.2, 94.9)
Total PBAC score		
<200	50/54 (92.6)<br>(82.1, 97.9)	57/58 (98.3)<br>(90.8, 100)
≥200	75/85 (88.2)<br>(79.4, 94.2)	71/83 (85.5)<br>(76.1, 92.3)
Birth experience		
No	62/69 (89.9)<br>(80.2, 95.8)	64/69 (92.8)<br>(83.9, 97.6)
Yes	63/70 (90.0)<br>(80.5, 95.9)	64/72 (88.9)<br>(79.3, 95.1)
Uterine volume (cm^3)		
≤170	46/50 (92.0)<br>(80.8, 97.8)	47/49 (95.9)<br>(86.0, 99.5)
>170	79/89 (88.8)<br>(80.3, 94.5)	81/92 (88.0)<br>(79.6, 93.9)
Uterine leiomyoma type		
Subserosal	50/58 (86.2)<br>(74.6, 93.9)	47/48 (97.9)<br>(88.9, 99.9)
Intramural	109/122 (89.3)<br>(82.5, 94.2)	108/120 (90.0)<br>(83.2, 94.7)
Submucosal	20/24 (83.3)<br>(62.6, 95.3)	27/33 (81.8)<br>(64.5, 93.0)
Cervical	3/3 (100)<br>(29.2, 100)	3/3 (100)<br>(29.2, 100)

PBAC, Pictorial Blood Loss Assessment Chart.
