# Supplementary material for: Linzagolix versus leuprorelin in Japanese women with uterine leiomyomas: a phase 3, randomized, active-controlled, non-inferiority trial
Source: Hum Reprod. 2026 Jul 6;41(9):1534–43. doi: 10.1093/humrep/deag106 (PMC13550677; doi:10.1093/humrep/deag106)
Supplement: Supplementary file 7 [file deag106_supplementary_table_s5.pdf]

*Human Reproduction*, 2026, 1–1
https://doi.org/10.1093/humrep/deag106
**Supplementary Data**

human reproduction

OXFORD

**Supplementary Table S5.** Proportion of patients with a maximum NRS score ≤1 over time (full analysis set).

| n/N (%) | Linzagolix (n = 68) | Leuprorelin (n = 69) | Difference vs leuprorelin, % (95% CI) |
|---|---|---|---|
| Days 1–28 | 15/68 (22.1) | 14/69 (20.3) | 1.8 (−12.1, 15.6) |
| Days 29–56 | 44/66 (66.7) | 48/68 (70.6) | −3.9 (−19.5, 11.7) |
| Days 57–84 | 47/64 (73.4) | 55/68 (80.9) | −7.4 (−21.8, 6.9) |
| 28 days prior to Week 12[a] | 49/68 (72.1) | 55/69 (79.7) | −7.7 (−21.9, 6.6) |
| Days 85–112 | 51/64 (79.7) | 51/67 (76.1) | 3.6 (−10.9, 18.1) |
| Days 113–140 | 47/64 (73.4) | 53/66 (80.3) | −6.9 (−21.4, 7.6) |
| Days 141–168 | 50/63 (79.4) | 54/64 (84.4) | −5.0 (−18.6, 8.6) |
| 28 days prior to end of treatment | 53/68 (77.9) | 58/69 (84.1) | −6.1 (−19.4, 7.1) |

Analyses included patients with pain symptoms at baseline (defined as a maximum NRS score ≥4 and an NRS score ≥1 for ≥2 days during the menstrual cycle preceding the treatment period).

[a] Defined as Days 57–84 for patients who reached Week 12, and 28 days prior to the end of treatment for patients who discontinued prior to Week 12.

NRS, numeric rating scale.
