# Supplementary material for: Linzagolix versus leuprorelin in Japanese women with uterine leiomyomas: a phase 3, randomized, active-controlled, non-inferiority trial
Source: Hum Reprod. 2026 Jul 6;41(9):1534–43. doi: 10.1093/humrep/deag106 (PMC13550677; doi:10.1093/humrep/deag106)
Supplement: Supplementary file 8 [file deag106_supplementary_table_s6.pdf]

Human Reproduction, 2026, 1–1
https://doi.org/10.1093/humrep/deag106
Supplementary Data

human reproduction

OXFORD

Supplementary Table S6. Secondary efficacy outcomes related to anaemia (full analysis set).

	Linzagolix (n = 143)	Leuprorelin (n = 144)
Patients with blood haemoglobin ≥12 g/dl, n/N (%)[a]		
Week 12	23/26 (88.5)	19/23 (82.6)
Week 24	24/26 (92.3)	20/22 (90.9)
Blood haematocrit (%), mean (SD)		
Baseline	38.1 (3.6)	37.8 (3.8)
Week 12	40.9 (2.8)	41.2 (2.8)
Week 24	41.1 (2.6)	41.5 (2.5)
Serum iron (µg/dl), mean (SD)		
Baseline	62.2 (43.8)	58.5 (39.4)
Week 12	89.3 (42.7)	87.4 (40.3)
Week 24	92.0 (39.5)	93.7 (34.3)
Serum ferritin (ng/ml), mean (SD)		
Baseline	26.1 (26.7)	24.4 (20.1)
Week 12	42.9 (40.7)	39.7 (34.2)
Week 24	49.2 (44.2)	44.7 (32.9)

[a] Analysis included patients with haemoglobin <12 g/dl at baseline and receiving oral iron supplementation.
