# Supplementary material for: Linzagolix versus leuprorelin in Japanese women with uterine leiomyomas: a phase 3, randomized, active-controlled, non-inferiority trial
Source: Hum Reprod. 2026 Jul 6;41(9):1534–43. doi: 10.1093/humrep/deag106 (PMC13550677; doi:10.1093/humrep/deag106)
Supplement: Supplementary file 9 [file deag106_supplementary_table_s7.pdf]

Human Reproduction, 2026, 1–1
https://doi.org/10.1093/humrep/deag106
Supplementary Data

human reproduction
OXFORD

Supplementary Table S7. Distribution of patients across PGIC categories at Weeks 12 and 24 (full analysis set).

	Linzagolix		Leuprorelin	
**n (%)**	**Week 12 (n = 136)**	**Week 24 (n = 132)**	**Week 12 (n = 137)**	**Week 24 (n = 131)**
Very much improved	36 (26.5)	54 (40.9)	37 (27.0)	50 (38.2)
Much improved	59 (43.4)	48 (36.4)	51 (37.2)	41 (31.3)
Minimally improved	22 (16.2)	10 (7.6)	25 (18.2)	24 (18.3)
No change	16 (11.8)	15 (11.4)	20 (14.6)	16 (12.2)
Minimally worse	1 (0.7)	1 (0.8)	4 (2.9)	0
Much worse	1 (0.7)	3 (2.3)	0	0
Very much worse	1 (0.7)	1 (0.8)	0	0

Patients rated their overall symptoms due to uterine leiomyomas as 'very much improved', 'much improved', minimally improved', 'no change', 'minimally worse', 'much worse', or 'very much worse', relative to their overall symptoms before the start of the run-in period. The distribution of patients across PGIC categories was not significantly different between treatment groups ($P = 0.547$ at Week 12 and $P = 0.513$ at Week 24, based on two-sample Wilcoxon tests).
PGIC, Patient Global Impression of Change.
