# Supplementary material for: Linzagolix versus leuprorelin in Japanese women with uterine leiomyomas: a phase 3, randomized, active-controlled, non-inferiority trial
Source: Hum Reprod. 2026 Jul 6;41(9):1534–43. doi: 10.1093/humrep/deag106 (PMC13550677; doi:10.1093/humrep/deag106)
Supplement: Supplementary file 10 [file deag106_supplementary_table_s8.pdf]

Human Reproduction, 2026, 1–1
https://doi.org/10.1093/humrep/deag106
Supplementary Data

human reproduction
OXFORD

Supplementary Table S8. Summary of pharmacodynamic outcomes (full analysis set).

	Linzagolix (n = 143)	Leuprorelin (n = 144)
Plasma oestradiol (pg/ml), mean (SD)		
Baseline[a]	59.6 (89.6)	52.3 (75.0)
Week 2	8.9 (17.3)	150.7 (381.1)
Week 12	10.5 (29.2)	9.1 (15.0)
Week 24	8.3 (15.0)	9.9 (24.7)
Week 28[b]	123.2 (98.5)	40.5 (62.2)
Serum FSH (mIU/ml), mean (SD)		
Baseline[a]	9.5 (7.5)	10.8 (9.3)
Week 2	5.2 (2.9)	3.0 (2.2)
Week 12	4.6 (3.2)	5.1 (2.1)
Week 24	4.8 (3.3)	5.3 (2.1)
Week 28[b]	7.4 (9.2)	6.8 (3.6)
Serum LH (mIU/ml), mean (SD)		
Baseline[a]	3.6 (3.2)	4.1 (3.7)
Week 2	1.0 (1.1)	2.2 (1.5)
Week 12	2.0 (4.1)	0.3 (0.4)
Week 24	1.9 (2.0)	0.2 (0.3)
Week 28[b]	6.0 (13.3)	1.3 (2.2)
Serum progesterone (ng/ml), mean (SD)		
Baseline[a]	0.3 (0.4)	0.3 (0.7)
Week 2	0.2 (0.4)	0.4 (1.3)
Week 12	0.2 (0.3)	0.2 (0.9)
Week 24	0.1 (0.1)	0.1 (0.0)
Week 28[b]	5.2 (7.2)	0.7 (3.2)

[a] Baseline pharmacodynamic tests were performed at the start of the treatment period, which occurred on Days 1–5 of menstruation.
[b] Week 28 represents Week 4 of the follow-up period.
