# Supplementary material for: Linzagolix versus leuprorelin in Japanese women with uterine leiomyomas: a phase 3, randomized, active-controlled, non-inferiority trial
Source: Hum Reprod. 2026 Jul 6;41(9):1534–43. doi: 10.1093/humrep/deag106 (PMC13550677; doi:10.1093/humrep/deag106)
Supplement: Supplementary file 11 [file deag106_supplementary_table_s9.pdf]

Human Reproduction, 2026, 1–1
https://doi.org/10.1093/humrep/deag106
Supplementary Data

human reproduction
OXFORD

Supplementary Table S9. Shift in bone density *T*-scores over time (safety set).

	Linzagolix				Leuprorelin			
**n (%)**	**Week 12 (n = 137)**	**Week 24 (n = 129)**	**End of treatment (n = 141)**	**Week 48[a] (n = 125)**	**Week 12 (n = 138)**	**Week 24 (n = 132)**	**End of treatment (n = 143)**	**Week 48[a] (n = 131)**
From >−1 at baseline to:								
>−1	120 (87.6)	106 (82.2)	118 (83.7)	111 (88.8)	119 (86.2)	105 (79.5)	115 (80.4)	105 (80.2)
>−2.5 and ≤−1	6 (4.4)	12 (9.3)	12 (8.5)	4 (3.2)	5 (3.6)	14 (10.6)	14 (9.8)	13 (9.9)
≤−2.5	0	0	0	0	0	0	0	0
From >−2.5 and ≤−1 at baseline to:								
>−1	0	0	0	0	0	0	0	0
>−2.5 and ≤−1	11 (8.0)	10 (7.8)	10 (7.1)	9 (7.2)	14 (10.1)	13 (9.8)	14 (9.8)	13 (9.9)
≤−2.5	0	1 (0.8)	1 (0.7)	1 (0.8)	0	0	0	0
From ≤−2.5 at baseline to:								
>−1	0	0	0	0	0	0	0	0
>−2.5 and ≤−1	0	0	0	0	0	0	0	0
≤−2.5	0	0	0	0	0	0	0	0

A *T*-score >−1 indicates normal bone density, a *T*-score >−2.5 and ≤−1 indicates osteopenia, and a *T*-score ≤−2.5 indicates osteoporosis.
[a] Week 48 represents Week 24 of the follow-up period.

© The Author(s) 2026. Published by Oxford University Press on behalf of European Society of Human Reproduction and Embryology.
This is an Open Access article distributed under the terms of the Creative Commons Attribution License (https://creativecommons.org/licenses/by/4.0/), which permits unrestricted reuse, distribution, and reproduction in any medium, provided the original work is properly cited.
Human Reproduction, 2026, 00, 1–21
https://doi.org/10.1093/humrep/deag106
Original Article
